# Supplementary material for: The platelet receptor CLEC-2 blocks neutrophil mediated hepatic recovery in acetaminophen induced acute liver failure
Source: Nat Commun. 2020 Apr 22;11:1939. doi: 10.1038/s41467-020-15584-3 (PMC7176690; doi:10.1038/s41467-020-15584-3)
Supplement: Supplementary file 1 — Supplementary Information [file 41467_2020_15584_MOESM1_ESM.pdf]

The platelet activating receptor CLEC-2 blocks neutrophil mediated hepatic recovery in acetaminophen induced acute liver failure

Chauhan et al

Supplementary Information:

Supplementary Figure 1

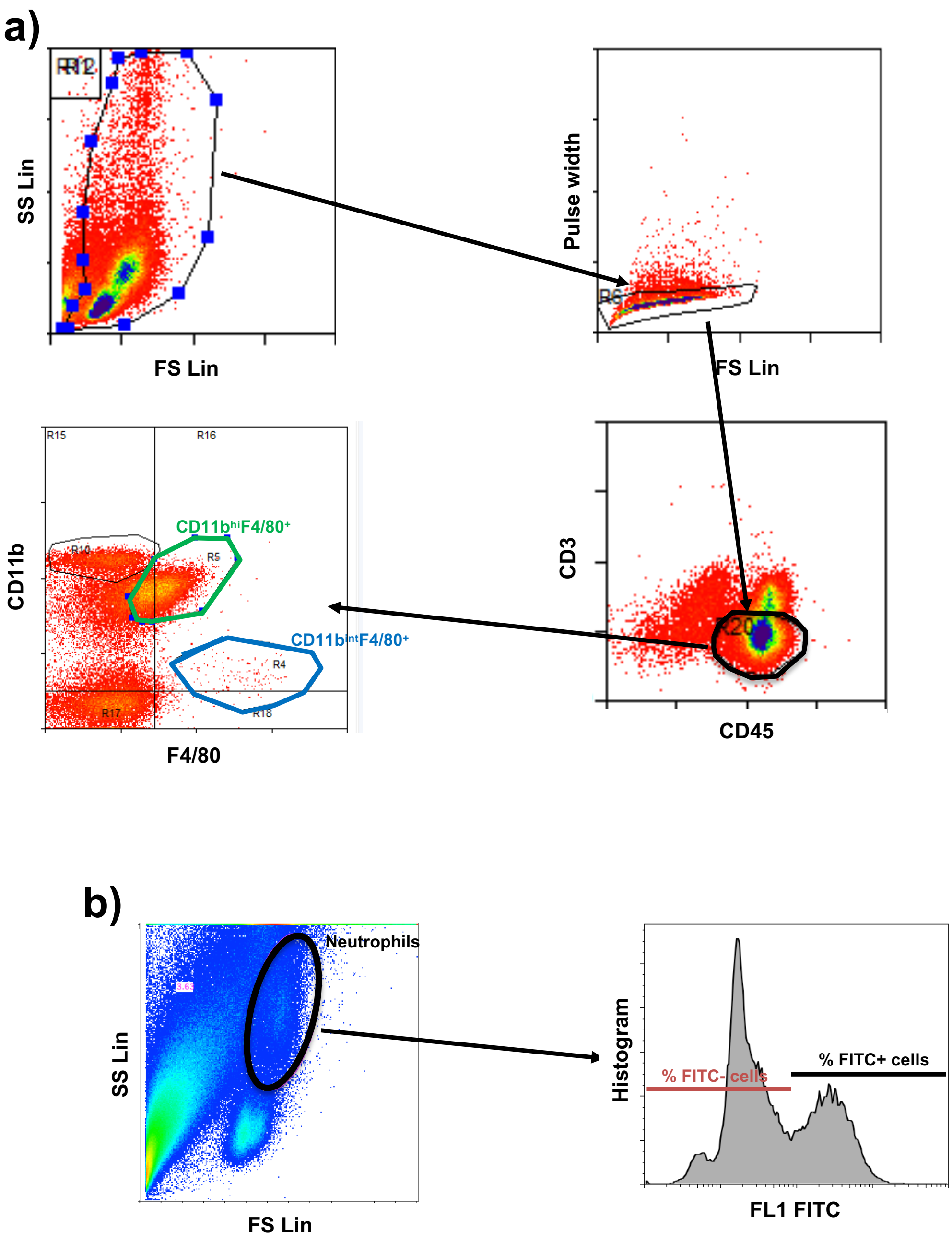

Supplementary Figure 1: Gating strategy employed to delineate infiltrating vs resident macrophage and percentage of neutrophils that had phagocytosed FITC^{+} bacteria

Gating strategy employed to a) delineate hepatic macrophages present after toxic liver injury into either Kupffer cells (CD45^{+}CD3^{-}CD11b^{int}F4/80^{+}) or infiltrating macrophages (CD45^{+}CD3^{-}CD11b^{hi}F4/80^{+}) (n=4). b) Neutrophil fraction from whole mouse blood was gated based on granularity (as per manufacturer's instructions) (left graph). Gating strategy for identifying percentage of neutrophils that were FITC positive after being incubated with FITC labelled bacteria is shown right graph) (n=3).

Supplementary Figure 2

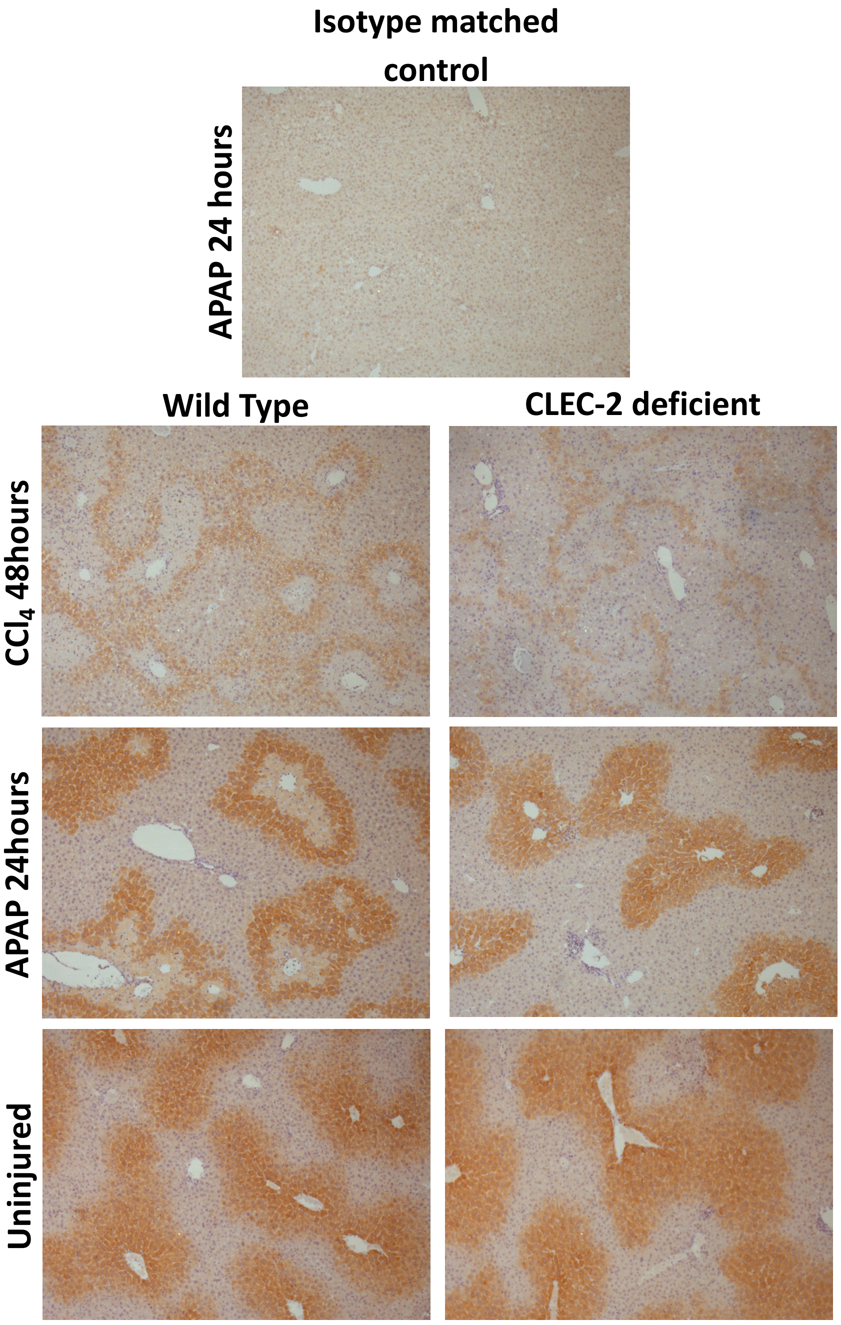

Supplementary Figure 2 CYP2E1 expression is not influenced by CLEC-2 deficiency

Uninjured and injured (with APAP or CCl_4) WT or CLEC-2 deficient *mice* were sacrificed at peak point of liver injury (48 hours CCl_4, 24hrs-APAP), or the corresponding time point for the uninjured control group. Mouse liver tissue was collected, and paraffin embedded. Stained with a CYP2E1 antibody (or isotype matched control) with a DAB (brown) secondary stain. Representatives images showed at 10x magnification. (n = 6 *mice*).

Supplementary Figure 3

Wild Type

CLEC-2 deficient

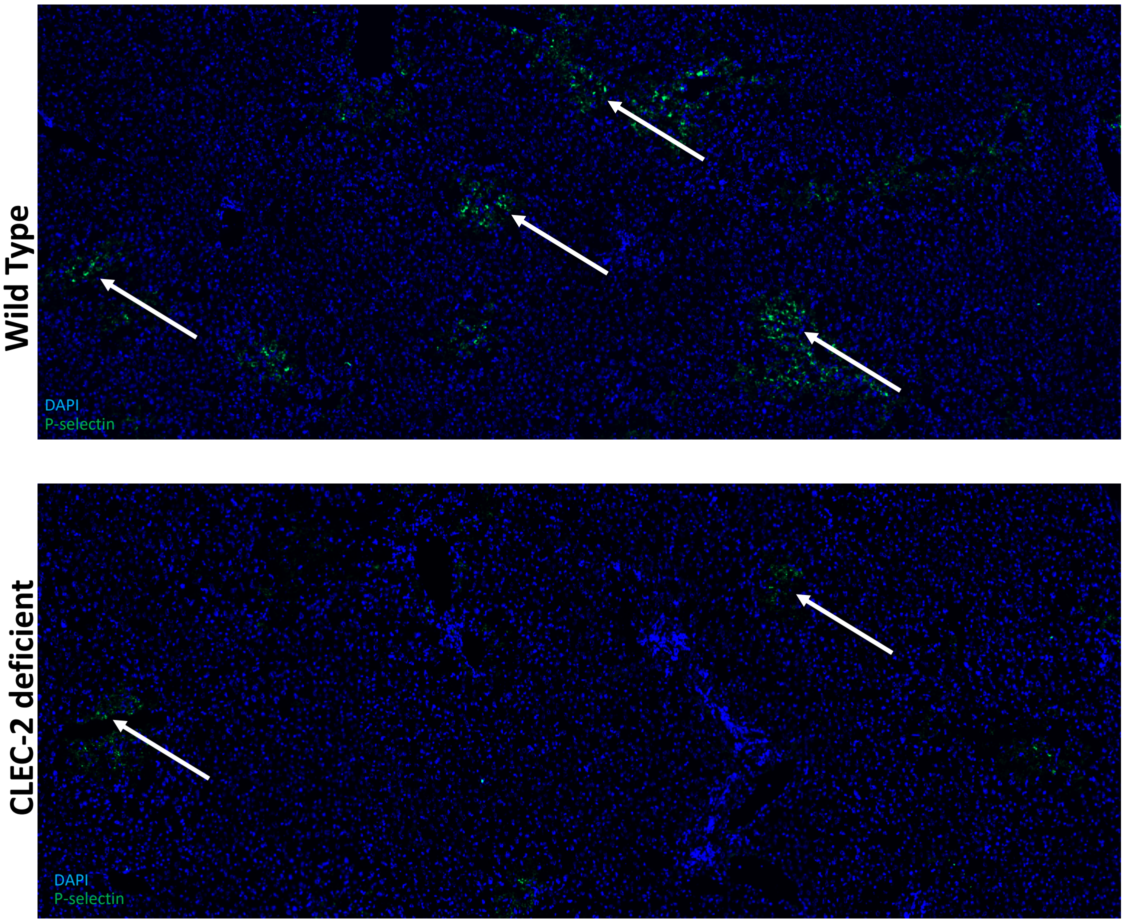

Supplementary Figure 3: CLEC-2 deficient *mice* have less P-selectin expression after toxic liver injury

Wide view pictures of tissue sections from injured murine livers demonstrating enhanced platelet numbers as gauged by P-selectin expression (white arrows) in wild type *mice* (top panel) compared to CLEC-2 deficient *mice* (bottom panel) (DAPI nuclear stain-blue, P-selectin-green). Acquired via Carl Zeiss AxioScan Z1 Slide Scanner using a 3CCD colour 2MP Hitachi 1200x1600 HV-F202SCL camera. Images were analysed using Zen blue (2012) slide scan software (10x magnification). Images are representative of 5 *mice*.

Supplementary Figure 4

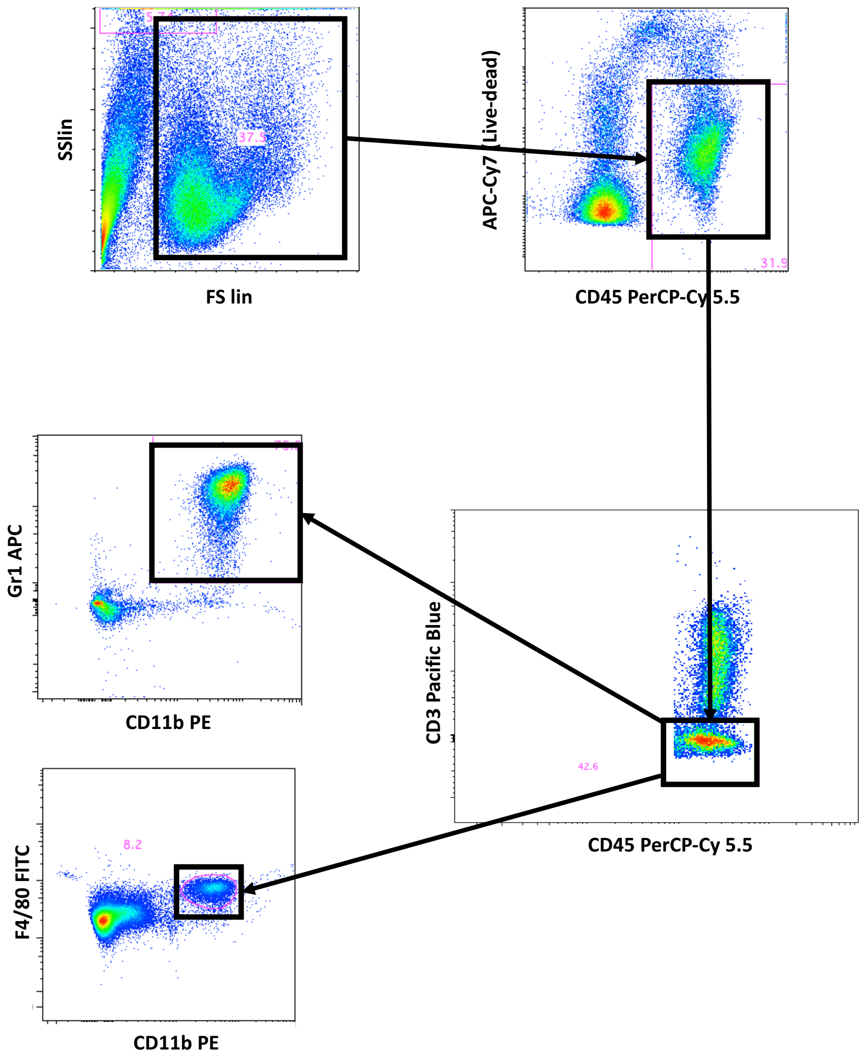

Supplementary Figure 4: Gating strategy employed to delineate cells of the myeloid lineage. After the live CD45$^+$ population was gated out (top right), gates were applied to separate out the CD3$^+$ population (bottom right figure). The CD3– population was then further gated into macrophages (CD45$^+$CD3$^-$CD11b$^+$F4/80$^+$) (bottom left) and neutrophils (CD45$^+$CD3$^-$CD11b$^+$GR1^{hi}) (Panel in the middle, left hand side).

Supplementary Figure 5

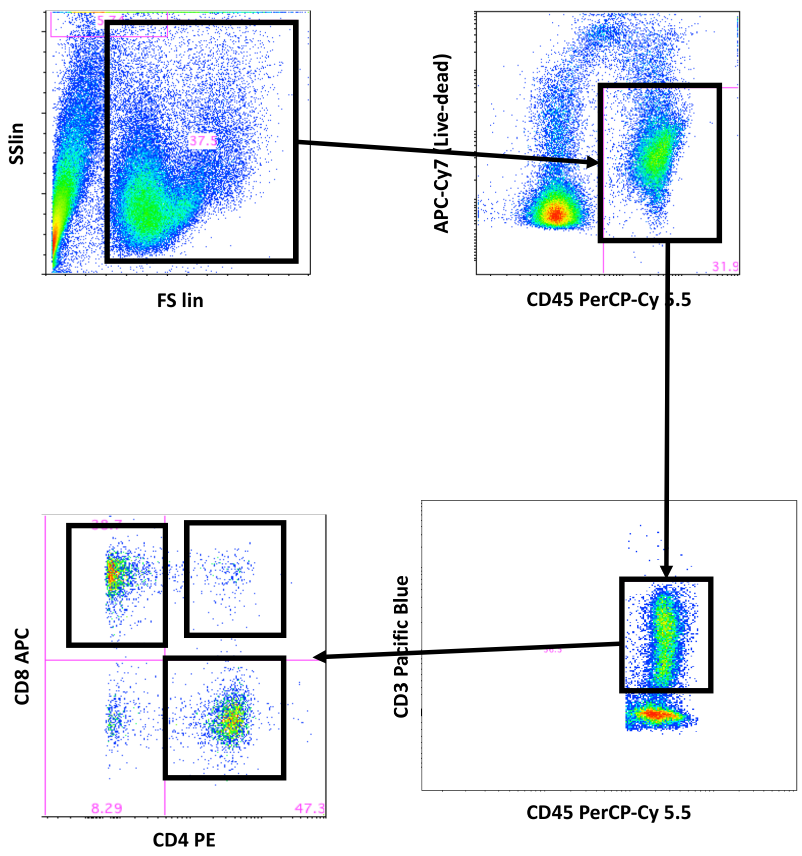

Supplementary Figure 5: Gating strategy employed to delineate lymphocytes. After the live CD45$^+$ population was gated out (top right), gates were applied to separate out the CD3- population (bottom right figure). The CD3$^+$ population was then further gated into CD8 T-cells (thus CD45$^+$CD3$^+$CD8$^+$CD4$^-$), CD4 T-cells (CD45$^+$CD3$^+$CD8$^-$CD4$^{+)}$ and CD3 T-cells (CD45$^+$CD3$^+$CD8$^-$CD4$^-$) (bottom left panel).

Supplementary Figure 6

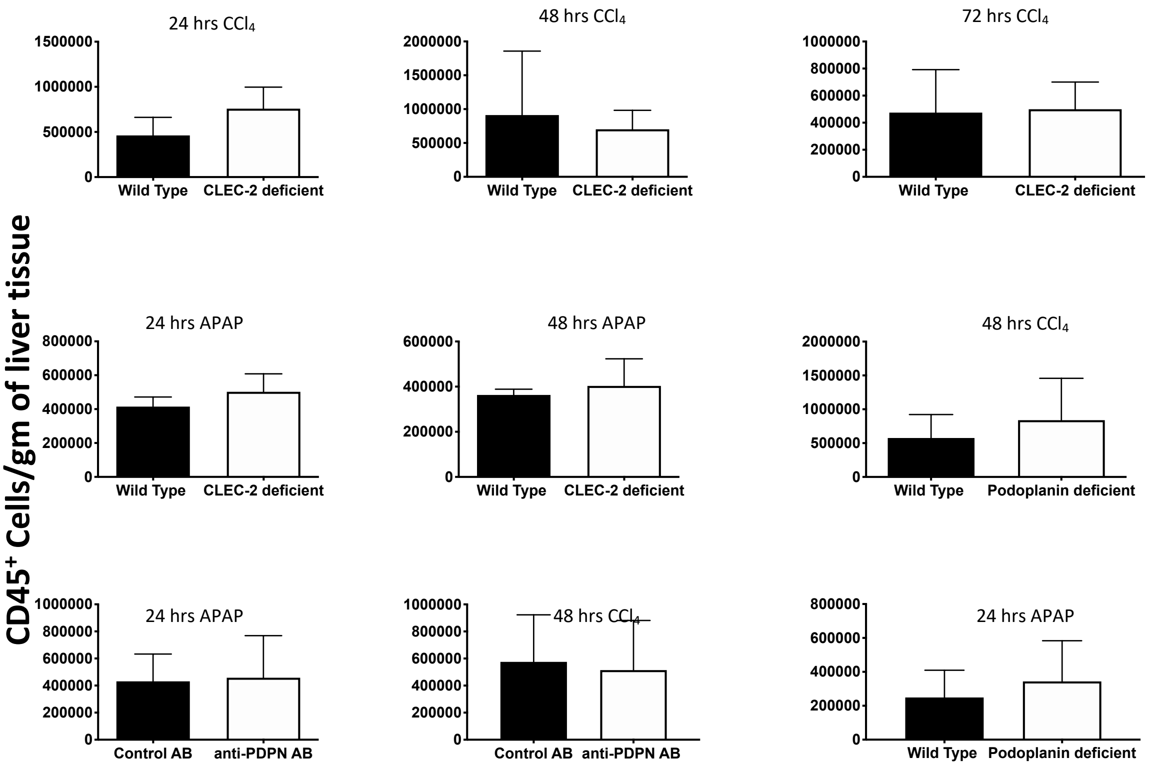

Supplementary Figure 6: Numbers of liver infiltrating CD45 + leukocytes are similar at most timepoints after toxic liver injury

WT or CLEC-2 deficient or podoplanin deficient or *mice* that had been pre-treated with an anti-podoplanin blocking antibody were injected with a single dose of IP CCl_4 or IP APAP. *Mice* were sacrificed at 24, 48 or 72 hours after injection. The liver was digested to isolate leucocytes, which were expressed as numbers of cells per gram of liver tissue. (*$P < 0.05$, **$P < 0.01$, ***$P < 0.001$). (n=4-10, data analyzed using either unpaired Students T-test or Mann-Whitney test).

Supplementary Figure 7

Ki67 staining

a)

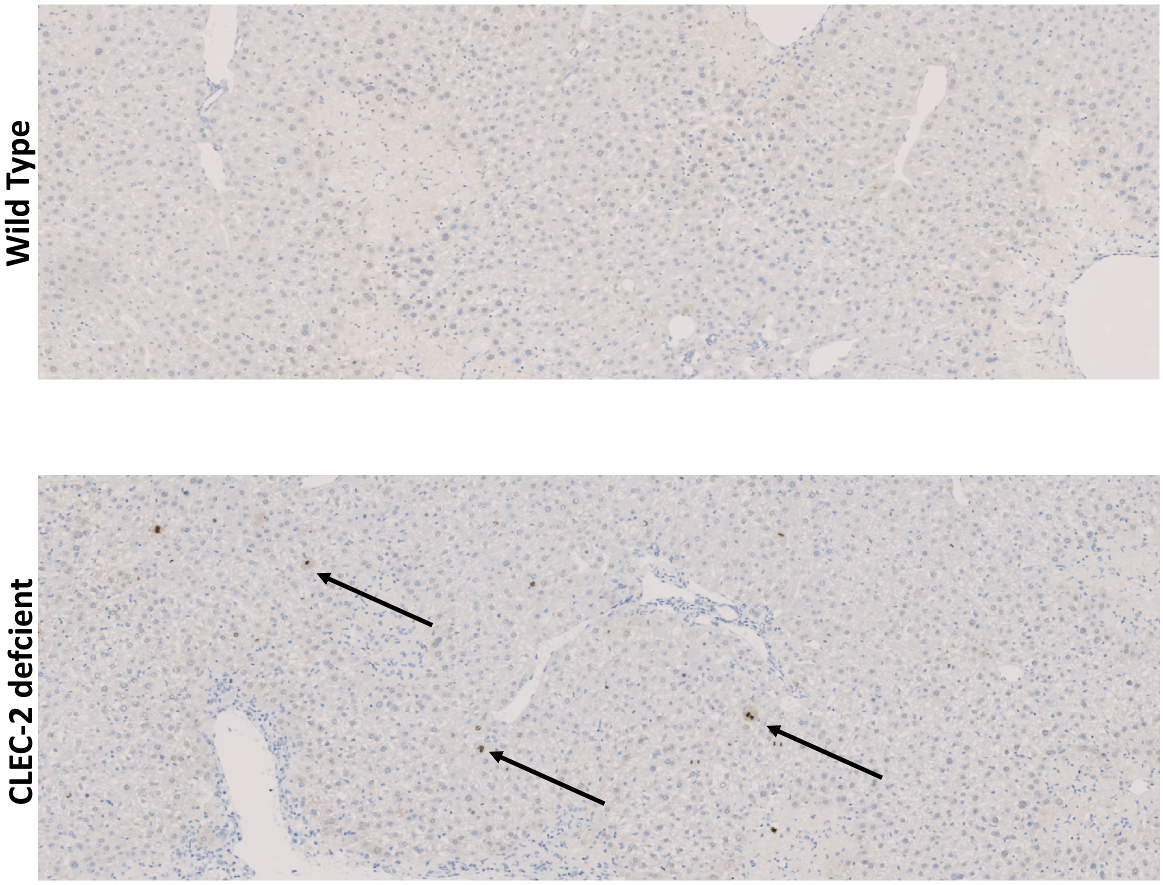

b)

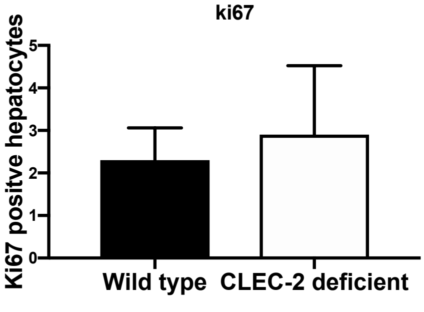

Supplementary Figure 7: Ki67 regeneration after toxic liver injury

Wide view brightfield pictures of tissue sections from injured murine livers demonstrating enhanced Ki67 expression in CLEC-2 deficient *mice* (black arrows-bottom panel) compared to wild type *mice* (top panel). Acquired via Carl Zeiss AxioScan Z1 Slide Scanner using a 3CCD color 2MP Hitachi 1200x1600 HV-F202SCL camera. Images were analysed using Zen blue (2012) slide scan software(10x magnification). Images are representative of 4 *mice*. b) Ki67 positive hepatocytes were counted from 10 randomly selected central fields in CLEC-2 deficient and WT *mice* after toxic liver injury.

Supplementary Figure 8

a)

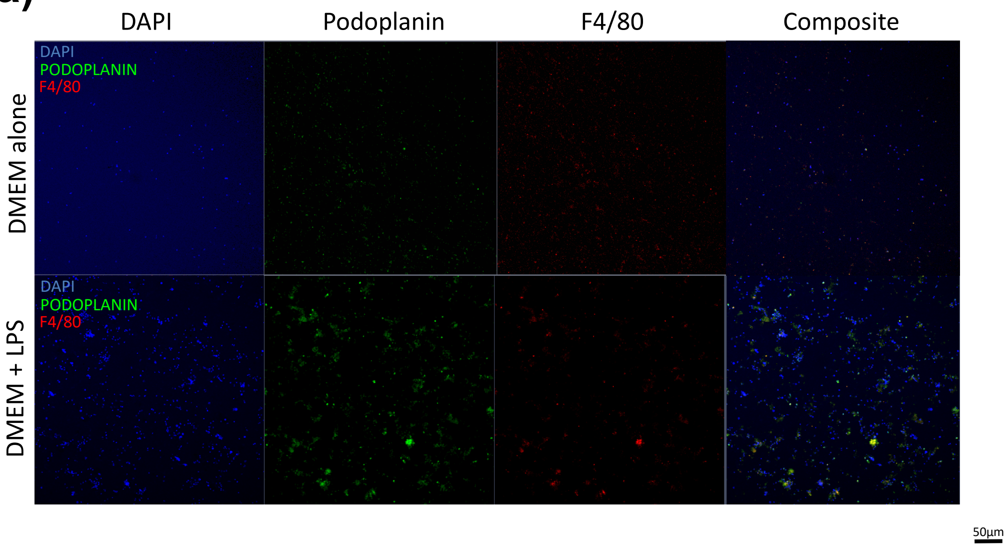

b)

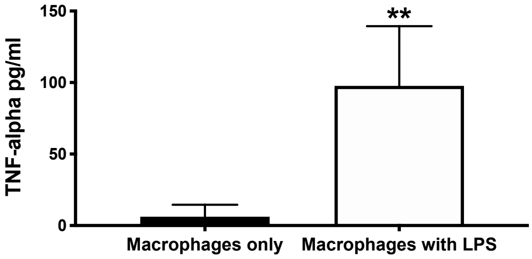

Supplementary Figure 8: Isolated macrophages upregulate podoplanin and produce TNF-α in vitro on stimulation with lipo-polysaccharide

Non-injured WT *mice* were sacrificed, their livers were removed and hepatic macrophages isolated. a) Confocal microscopy images (10x) confirming the isolated population containing F4/80+ cells (red), which express little podoplanin (green) at rest (DMEM alone), but macrophages from the same mouse dramatically upregulate podoplanin on stimulation with LPS containing media. b) After being stimulated with LPS (or control DMEM alone, the macrophage secretome was collected and analyzed via a sandwich ELISA. (*P < 0.05, **P < 0.01, ***P < 0.001; n = 3-6, data analyzed using unpaired Students T-test).

Supplementary Figure 9

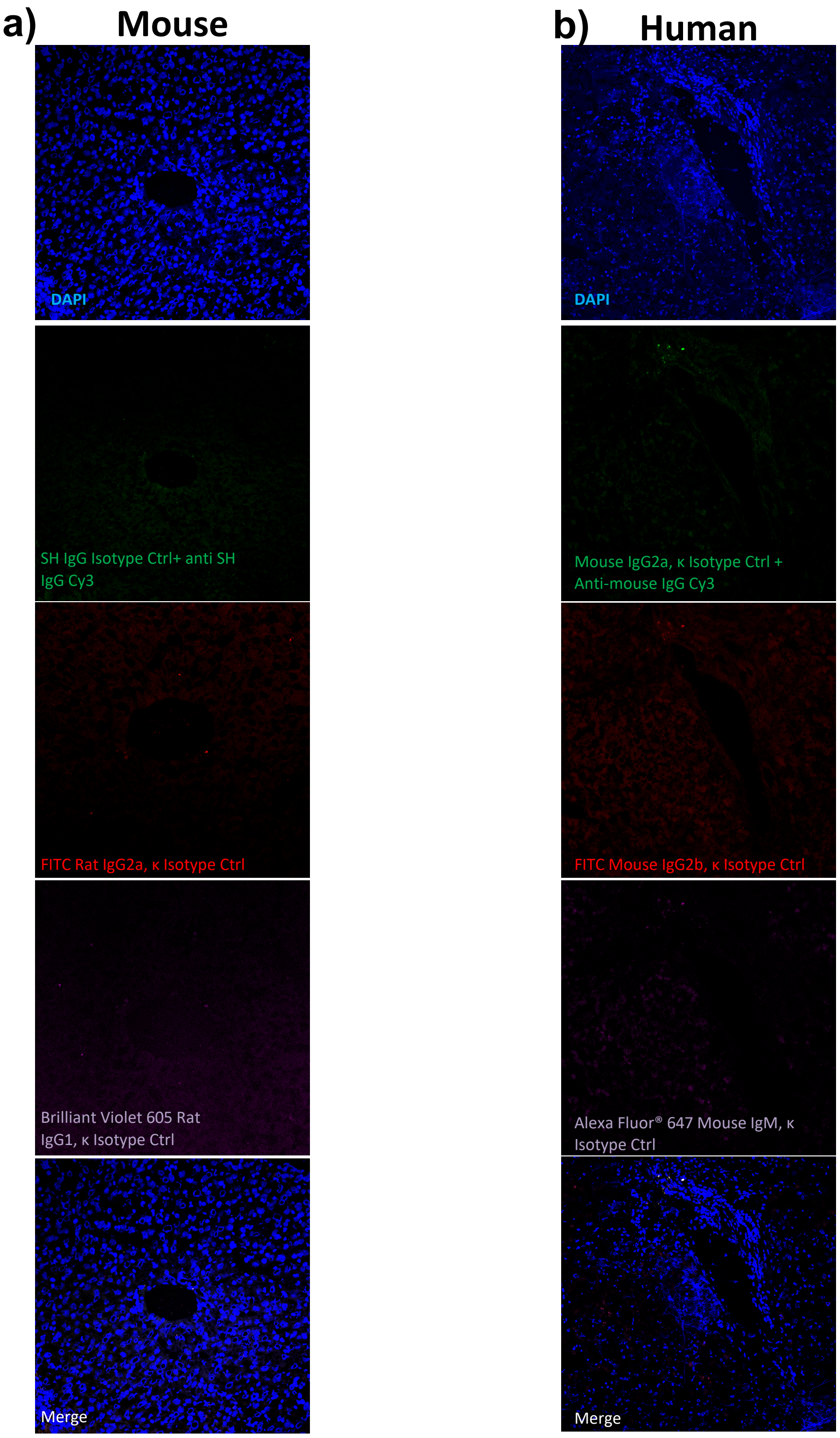

Supplementary Figure 9: Isotype control matched antibody staining for Figures 1 and 2

Representative control antibody stainings are shown for both a) murine sections and b) human sections after a toxic liver injury 10x magnification. Images are representative of 5 samples in *humans* and 6 in *mice*.
